# A fuzzy rank-based ensemble of CNN models for classification of cervical cytology

**DOI:** 10.1038/s41598-021-93783-8

**Published:** 2021-07-15

**Authors:** Ankur Manna, Rohit Kundu, Dmitrii Kaplun, Aleksandr Sinitca, Ram Sarkar

**Affiliations:** 1 Department of Computer Science and Engineering, Jadavpur University, Kolkata, 700032 India; 2 Department of Electrical Engineering, Jadavpur University, Kolkata, 700032 India; 3 Department of Automation and Control Processes, Saint Petersburg Electrotechnical University “LETI”, Saint Petersburg, 197376 Russian Federation

**Keywords:** Cancer imaging, Cancer imaging

## Abstract

Cervical cancer affects more than 0.5 million women annually causing more than 0.3 million deaths. Detection of cancer in its early stages is of prime importance for eradicating the disease from the patient’s body. However, regular population-wise screening of cancer is limited by its expensive and labour intensive detection process, where clinicians need to classify individual cells from a stained slide consisting of more than 100,000 cervical cells, for malignancy detection. Thus, Computer-Aided Diagnosis (CAD) systems are used as a viable alternative for easy and fast detection of cancer. In this paper, we develop such a method where we form an ensemble-based classification model using three Convolutional Neural Network (CNN) architectures, namely Inception v3, Xception and DenseNet-169 pre-trained on ImageNet dataset for Pap stained single cell and whole-slide image classification. The proposed ensemble scheme uses a fuzzy rank-based fusion of classifiers by considering two non-linear functions on the decision scores generated by said base learners. Unlike the simple fusion schemes that exist in the literature, the proposed ensemble technique makes the final predictions on the test samples by taking into consideration the confidence in the predictions of the base classifiers. The proposed model has been evaluated on two publicly available benchmark datasets, namely, the SIPaKMeD Pap Smear dataset and the Mendeley Liquid Based Cytology (LBC) dataset, using a 5-fold cross-validation scheme. On the SIPaKMeD Pap Smear dataset, the proposed framework achieves a classification accuracy of 98.55% and sensitivity of 98.52% in its 2-class setting, and 95.43% accuracy and 98.52% sensitivity in its 5-class setting. On the Mendeley LBC dataset, the accuracy achieved is 99.23% and sensitivity of 99.23%. The results obtained outperform many of the state-of-the-art models, thereby justifying the effectiveness of the same. The relevant codes of this proposed model are publicly available on GitHub.

## Introduction

Cervical Cancer is the fourth most common category of cancer in women, affecting more than 0.5 million women worldwide and causing more than 0.3 million deaths annually. Hence, early detection is crucial for preventing and curing this cancer. The primary limitation in the diagnosis of cervical cancer is the complex and time-consuming detection procedure, which requires experts to classify each cell from a slide containing more than 100,000 cervical cells stained by the Papanicolaou method by Gill et al.^1^. Besides, the subjective variability in the screening process may lead to fatal errors in the diagnosis. Such a labour-intensive and expensive procedure prohibits the population-wise screening of cervical cancer, especially in underdeveloped and developing countries. So, the researchers have been trying to develop many automated Computer-Aided Diagnosis (CAD) methods for the fast, sensitive and accurate detection of cervical cancer, which can augment the success of pathologists and doctors in cancer diagnosis and prevention.

Deep learning^2^ is an important tool of Artificial Intelligence (AI) that has been prevalent in formulating decision-support systems for biomedical image classification^3^. However, end-to-end classification using deep learning models requires a lot of training data to provide satisfactory performance, which is often not available in the medical domain. Transfer learning is one of the solutions to this problem, where a model trained on a dataset containing a very large amount of data is re-used (sometimes after re-training) in the present problem with the small dataset. However, different models might predict well on certain distributions of data, that is, the classification in some classes in the dataset might be more accurate than the others. Besides, conventional rank based ensemble techniques does not utilize the distribution of the prediction probabilities. As a result, important information may remain unused. Keeping this fact in mind, in this work, we propose a novel approach where we utilize all the information available from different base learners by quantifying two important parameters—the closeness of the prediction probability to 1 and deviation of the prediction probability from 1. Moreover, our approach fuses all such quantified values for making the final prediction so that it can deal with the classification problem under consideration more effectively and make a fairly accurate prediction.

Ensemble learning is one such alternative where decision scores from multiple classifiers are fused to predict the final class label of an input sample. An ensemble model is aimed to capture the salient features of all its constituent models thus performing better than the individual base classifiers. Such models are robust since ensembling diminishes the dispersion or spread of the predictions made by the base models. The variance in the prediction errors of the base classifiers gets reduced in the ensemble model by the addition of some bias to the competing base learners.

In the present work, we formulate a fusion strategy that uses the decision scores obtained by three base Convolutional Neural Network (CNN) classifiers, namely, Inception v3 by Szegedy et al.^4^, Xception by^5^ and DenseNet-169 by Huang et al.^6^ (pre-trained on the ImageNet dataset^7^) to form the ensemble. We use a fuzzy ranking-based approach, where the probability scores are subjected to two non-linear functions, an exponentially decaying function, and the *tanh* function, to assign the ranks to the class probabilities predicted by a base learner. The ranks assigned by the two non-linear functions are multiplied. The same process is repeated for each base learner, and the rank products from each classifier are added to get the final ranks. We use two different functions of different concavities so that they can generate complementary results. Fusion entails consolidating the multiple ranks associated with an identity and determining a new rank that would aid in establishing the final decision. The main motive of using two ranks is to consider the closeness to and deviation from the expected result corresponding to the primary classification result. Lesser deviation corresponds to a lower value of the product and a better result. So, the class having the lowest value of this sum of products of ranks is deemed as the predicted class of the ensemble model. Here, the two non-linear functions have opposite concavity in the range [0, 1] and hence a higher confidence score results in a larger value of rank in one function and a smaller value in the other, and our aim to minimize this product. If the confidence score of a prediction is high, then this sum of products yields a lower value than if the confidence score is low which are explained in detail later.

**Figure 1:**
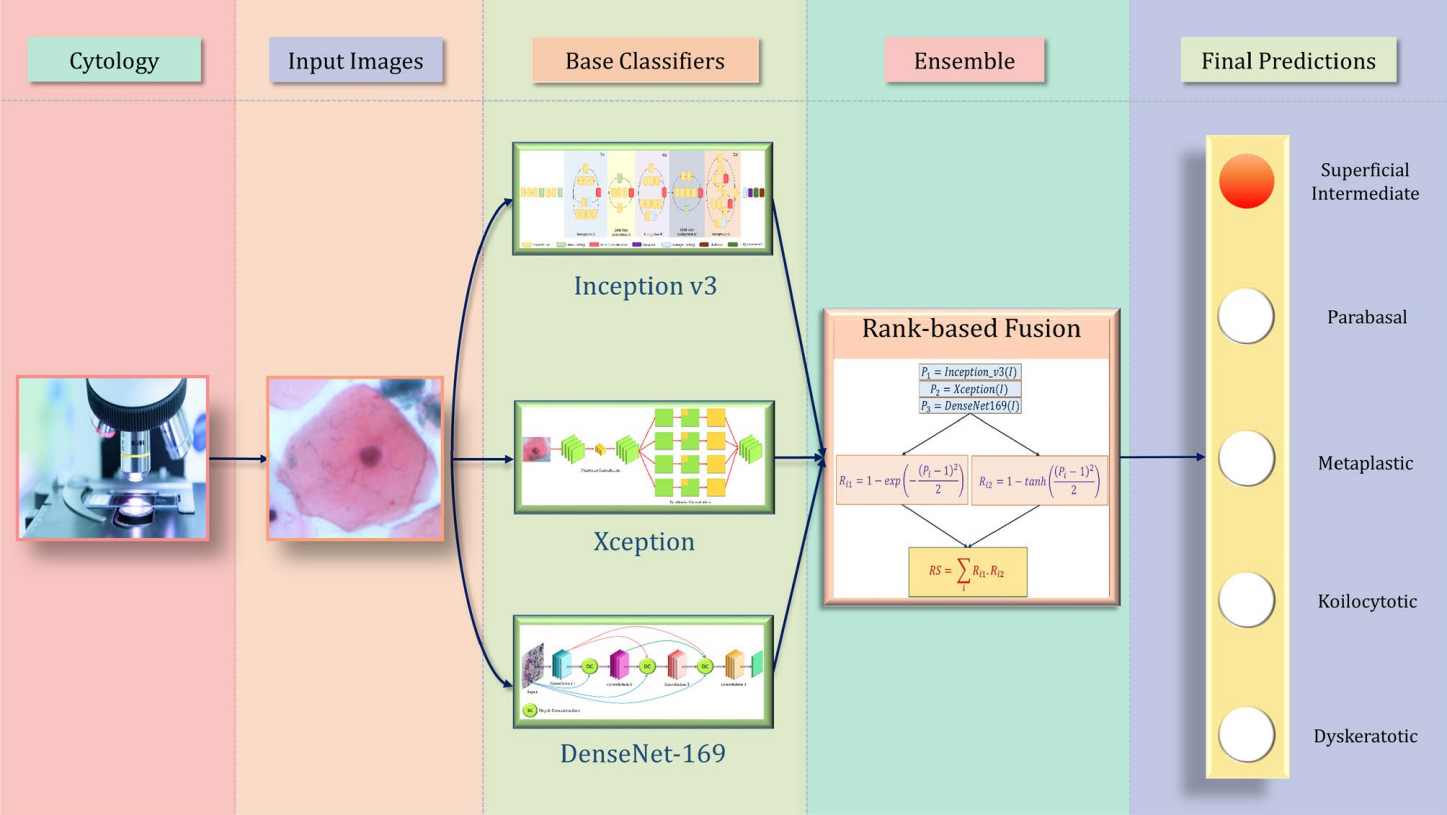
Overall structure of the proposed fuzzy rank-based ensemble of CNN models used for classification of cervical cytology (the image of the microscope under “Cytology” has been taken from the website (open-access) by Marsh et al.^8^, and the pap stained image under “Input Images” has been taken from the publicly available SIPaKMeD Pap Smear dataset^9^ used in this research and the complete image has been made by R.K. using Google Slides).

Several methods have been developed over the years for the automatic classification of cervical cancer using cytology images. Traditional machine learning-based methods^10–12^, although computationally less complex, require extraction of handcrafted features, and feature selection for classification. This limits the performance of such models because of the two main reasons: (1) extraction of handcrafted features becomes difficult for complex data pattern, and (2) all these features may not be sufficiently informative, thus adversely affecting the model’s performance.

However, Win et al.^13^’s method yielded commendable performance. They used a shape-based iterative method for nuclei detection followed by employing a marker-control watershed approach for separating overlapping cytoplasm. The authors performed feature extraction from these segmented nuclei and used a Random Forest classifier for feature selection. They achieved a classification accuracy of 94.09% on the SIPaKMeD dataset by Plissiti et al.^9^ by ensembling traditional classifiers like Linear Discriminant Analysis (LDA), and Support Vector Machine (SVM), etc.

Deep learning-based methods can avoid the aforementioned limitations of traditional machine learning techniques in the following ways: (1) deep learning models perform end-to-end classification without the need for feature engineering; (2) self-learning is induced in these models, thereby making the models effective to learn complex patterns in datasets. CNNs are prevalent for classifying image data, for example, Zhang et al.^14^ performed end-to-end classification using a deep CNN architecture and evaluated their method on the HErlev dataset achieving an accuracy of 98.3%. CNN models learn to extract invariant features automatically using the convolution of image and filters, have translational invariance, and they perform better than machine learning or image processing methods, making them popular. However, deep learning models require a large amount of labelled data for producing satisfactory results, but such large volumes of medical data are difficult to acquire since experts (doctors or pathologists) are needed to classify the acquired data. So a popular concept, called transfer learning is used where a deep learning model trained on a large dataset is re-used for classification on the current data. Li et al.^15^ performed transfer learning using the Inception v3 deep CNN model on a cervical immunohistochemistry image dataset and obtained only 77.3% accuracy.

Ensemble learning is a strategy that considers decisions obtained from more than one model for making the final decision. Some simple fusion schemes have been explored in literature like Sarwar et al.^16^ who used an average probability-based ensemble and Xue et al.^17^ who used a majority voting based ensemble technique. However, such simplistic ensemble models do not take into account the confidence of predictions and use pre-determined or fixed weights associated with the base learners. Keeping this in mind, in this research, we propose a novel ensemble technique which fuses the decision scores from three base CNN based classifiers, namely Inception v3^4^, Xception^5^ and DenseNet-169^6^ while taking into account the confidence in predictions of the base learners.

### Motivation and contributions

The tedious detection process of cervical cancer makes it impossible to conduct regular screening throughout the population. In this paper, we propose an automated screening framework that is both accurate and time-efficient. Since the data available in the biomedical domain is scarce, an end-to-end classification system using purely deep learning methods may fail to perform satisfactorily on unseen data. So, we use three transfer learning-based CNN classifiers to form an ensemble model where the predictions from multiple competing models are taken into account. Although simple fusion schemes like majority voting, weighted averaging, etc., have been used in literature, they do not consider the confidence in the predictions of a classifier while computing the predictions. In the proposed method, we develop a mathematical model that considers this, thus achieving superior classification performance than conventionally used simple ensemble methods. The overall workflow of the framework is shown in Fig. 1.

The contributions of the current research work are as follows:

1. Ensemble learning using three bases learners namely, Inception v3^4^, Xception^5^ and DenseNet-169^6^ has been implemented that boosts the performance of the overall model for making predictions on the scarce available data.
2. The proposed ensemble method applies two non-linear functions of different concavities to determine the fuzzy ranks of the classes in the decision scores. The sum of products of the ranks of the three base learners are computed and the lower rank is attributed as the predicted class. The use of two non-linear functions ensures that the confidence in the predictions of the classifiers is accounted for in the computation of the ranks, thereby leading to superior predictions.
3. The way we quantify the deviation of the predicted value from the expected value is novel. Also, the boost in accuracy brought by proposed ensemble model is noteworthy.
4. The proposed framework outperforms many state-of-the-art methods on two benchmark cervical cytology image datasets: the SIPaKMeD Pap Smear dataset by Plissiti et al.^9^ and the Mendeley Liquid Based Cytology (LBC) dataset by Hussain et al.^18^ in terms of classification accuracy and sensitivity.
5. To justify the robustness in performance of the proposed ensemble framework, it has been tested on an additional multi-class medical image dataset: the Zenodo 5K dataset and the results obtained prove the superiority of the ensemble approach.

## Proposed method

In this section, we give a brief overview of the base learners we use and the necessary customization we apply to the basic models, followed by the implementation detail of the proposed fuzzy rank based fusion of confidence scores of the base learners. Here our motive for ensembling is to utilize each of the confidence factors generated from base learners fully by mapping them into non-linear functions. One of the mapped values signifies the abidance or closeness to 1 and the other one signifies the deviation from 1. This proposed approach overcomes the shortcoming of the conventional ranking methods which do not consider the fact mentioned above^19,20^, and this may lead to an incorrect result. In the present study, we use three base learners and evaluate our method on bio-medical image datasets. Initially, we train the base learners (customization with pre-trained models trained on ImageNet^7^) and take the confidence scores. After that, we map the scores on two different functions having different concavities to generate non-linear fuzzy ranks and generate a fused score by combining these two ranks, which helps us to quantify the total deviation from expected. Lesser the deviation shows better confidence towards a particular class. The class having the lowest deviation value is considered as the winner and is assigned as the final class value. Here, we first give a brief overview of the pre-trained CNN models used as base learners.

### Inception v3

**Figure 2:**
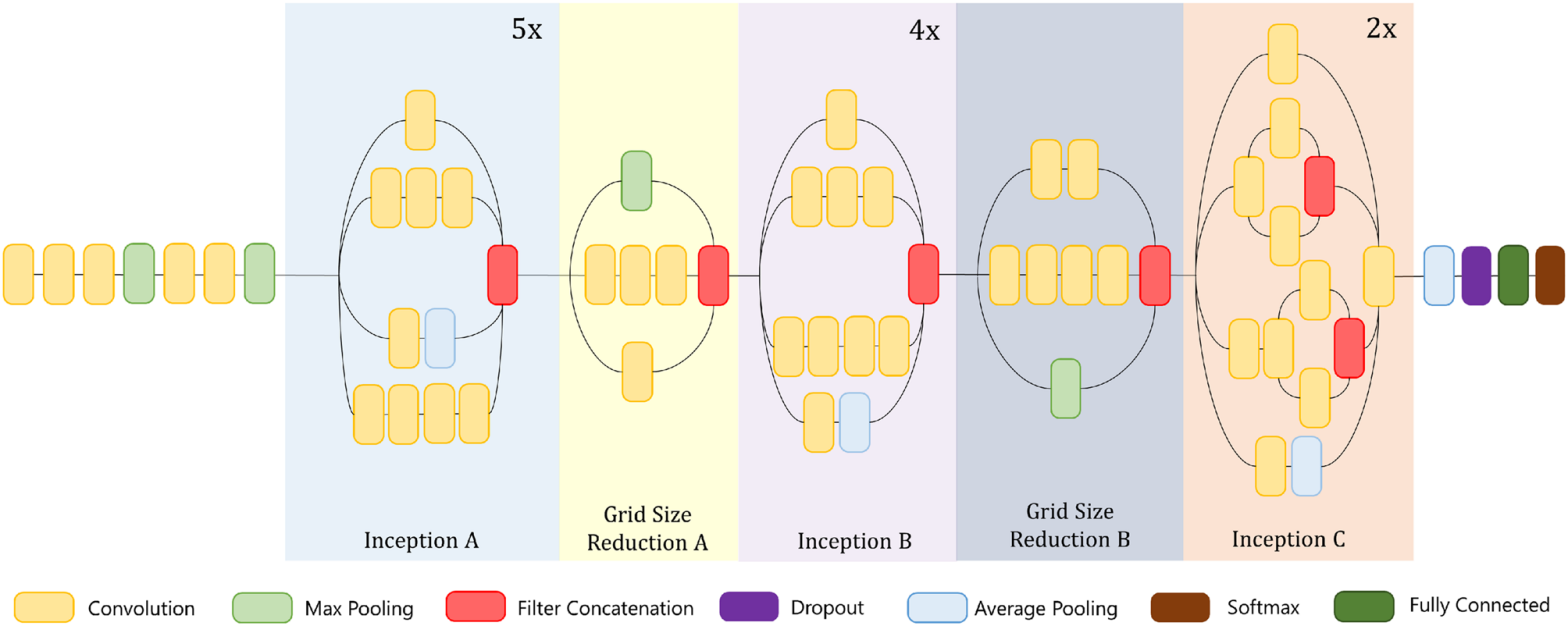
The architecture of the Inception v3 model: base learner 1 (image has been made by R.K. using Google Slides).

The most salient feature of the Inception v3 architecture developed by Szegedy et al.^4^ is the numerous parallel convolutions supported by the structure. This allows deep features to be generated while controlling the overfitting problem while using lesser computation than monolithic architectures like VGG-19. Figure 2 shows the architectural diagram of the Inception v3 CNN model.

### Xception

**Figure 3:**
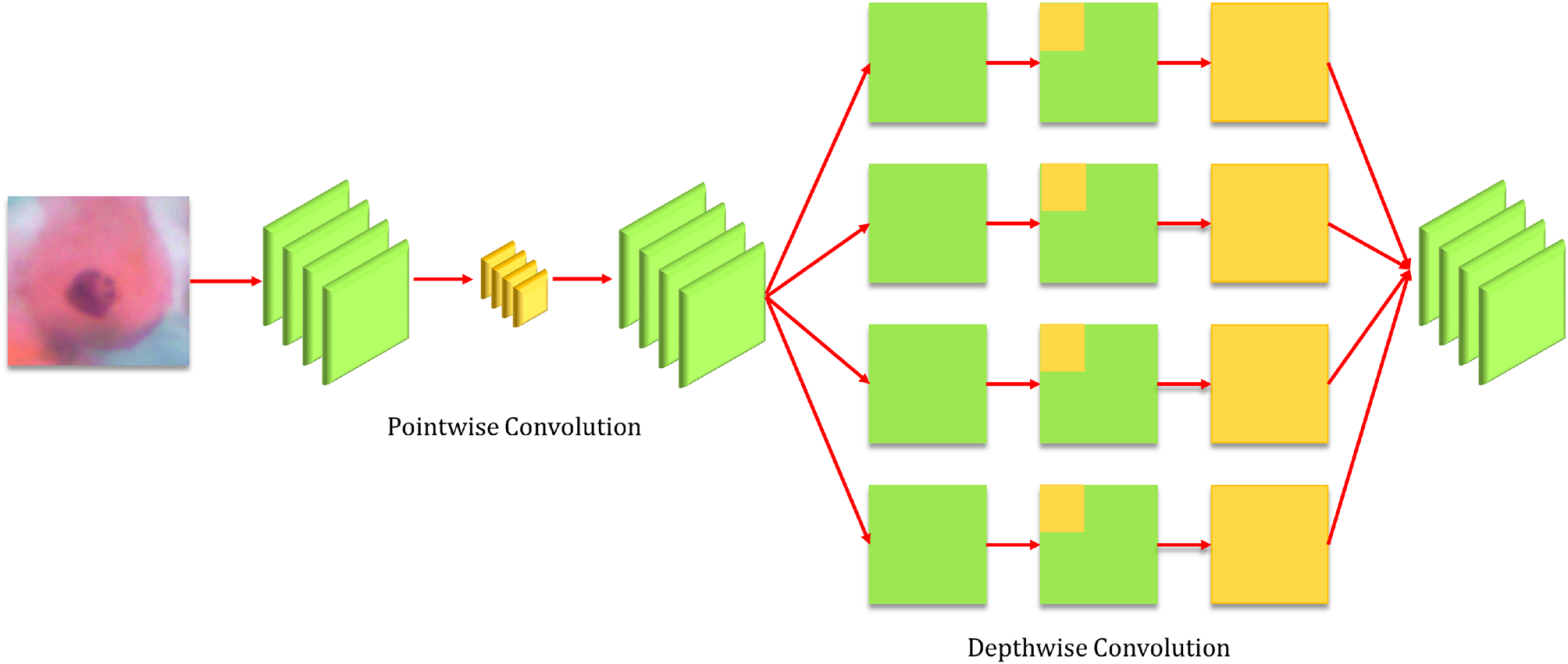
The architecture of the Xception model: base learner 2 (image has been made by R.K. using Google Slides).

The Xception architecture developed by Chollet et al.^5^ has been inspired from the Inception v3 architecture, consisting of the same number of model parameters as the latter, but the Xception architecture uses them more efficiently. They showed that pointwise convolutions and depthwise separable convolutions lie at the two extremes of a discrete spectrum, where the inception modules lie in the middle. Thus, they replaced the inception modules with depthwise separable convolutions, which provided a boost in the classification performance while incurring the same computation cost. The basic structure of the Xception model is shown in Fig. 3.

### DenseNet-169

**Figure 4:**
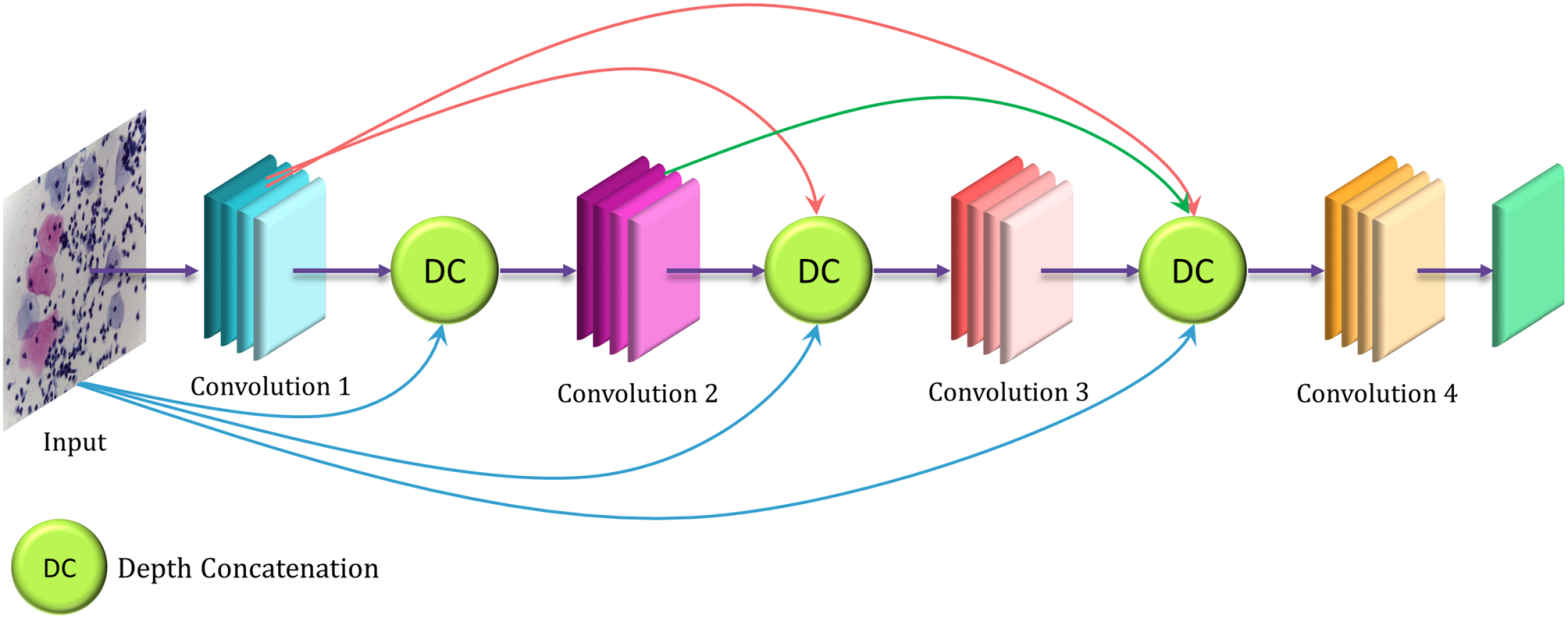
Architecture of the DenseNet model: base learner 3 (image has been made by R.K. using Google Slides).

The DenseNet architectures by Huang et al.^6^ are distinctive, in the sense that they provide a rich feature representation while also computationally efficient. The reason for that is, each layer in the DenseNet model is a concatenation of the feature maps in the current layer and all its preceding layers, as shown in Fig. 4. This makes the model compact since fewer channels are accommodated in the convolutional layers thus decreasing the number of trainable parameters, and the concatenation of the feature maps from the previous layers gives enhanced feature representation.

### Cascade of pre-trained model and customized layers

For better utilization of the information generated by pre-trained models, we add some customized layers based on the structure of the models. Next to the pre-trained models, we add a fully connected layer of 1024, 1028 and 256 nodes for Inception v3, DenseNet-169 and Xception respectively. This fully connected layer is associated with the Rectified Linear Unit (ReLU) activation function to overcome the vanishing gradient problem and faster learning. Then a dropout layer of 20% is added to avoid the problem of overfitting. If we directly calculate the confidence scores from such a high number of hidden units, we may lose some important information. To address this issue, at first, we cluster the necessary information into a lesser number of hidden nodes such as 128, 64, and 32 nodes for Inception v3, DenseNet-169 and Xception respectively. Then at the end, we implement class number specific output units. The hyperparameters used for training the CNN models have been set through extensive experiments and are shown in Table 1. The number of epochs used for fine-tuning the datasets has been set to 20, because the model weights are already optimized for image classification through pre-training on the ImageNet data, and we only need to train the customized layers that have been added to the CNN models, while keeping the weights of the other (pre-trained) layers fixed.

**Table 1:** Values of the hyperparameters used for training the base CNN classifiers.

Hyperparameter	Value
Optimizer	RMSProp
Loss function	Categorical cross entropy
Learning rate	2.00E−05
Batch size	32
Dropout rate	20%
Number of epochs	20

### Proposed ensemble approach

In this section, we detail the mathematical formulation for the proposed ensemble method. Let the confidence scores for C number of classes given by base learner i are ($P_1^i$, $P_2^i$, $P_3^i$, $...$, $P_C^i$), here i = 1, 2, 3. At first, we accumulate all the confidence scores obtained from each of the base learners. As ($P_1^i$, $P_2^i$, $P_3^i$, $...$, $P_C^i$) represent probabilities, essentially it will follow Eq. (1).

1 $$\begin{aligned} \sum _{k=1}^{C}P_k^i = 1, \,\,\forall \,\, i = 1,2,3. \end{aligned}$$

Let ($R_1^{i_1}$, $R_2^{i_1}$, $R_3^{i_1}$, $...$, $R_C^{i_1}$) and ($R_1^{i_2}$, $R_2^{i_2}$, $R_3^{i_2}$, $...$, $R_C^{i_2}$) are fuzzy ranks generated by using the two non-linear functions.

**Figure 5:**
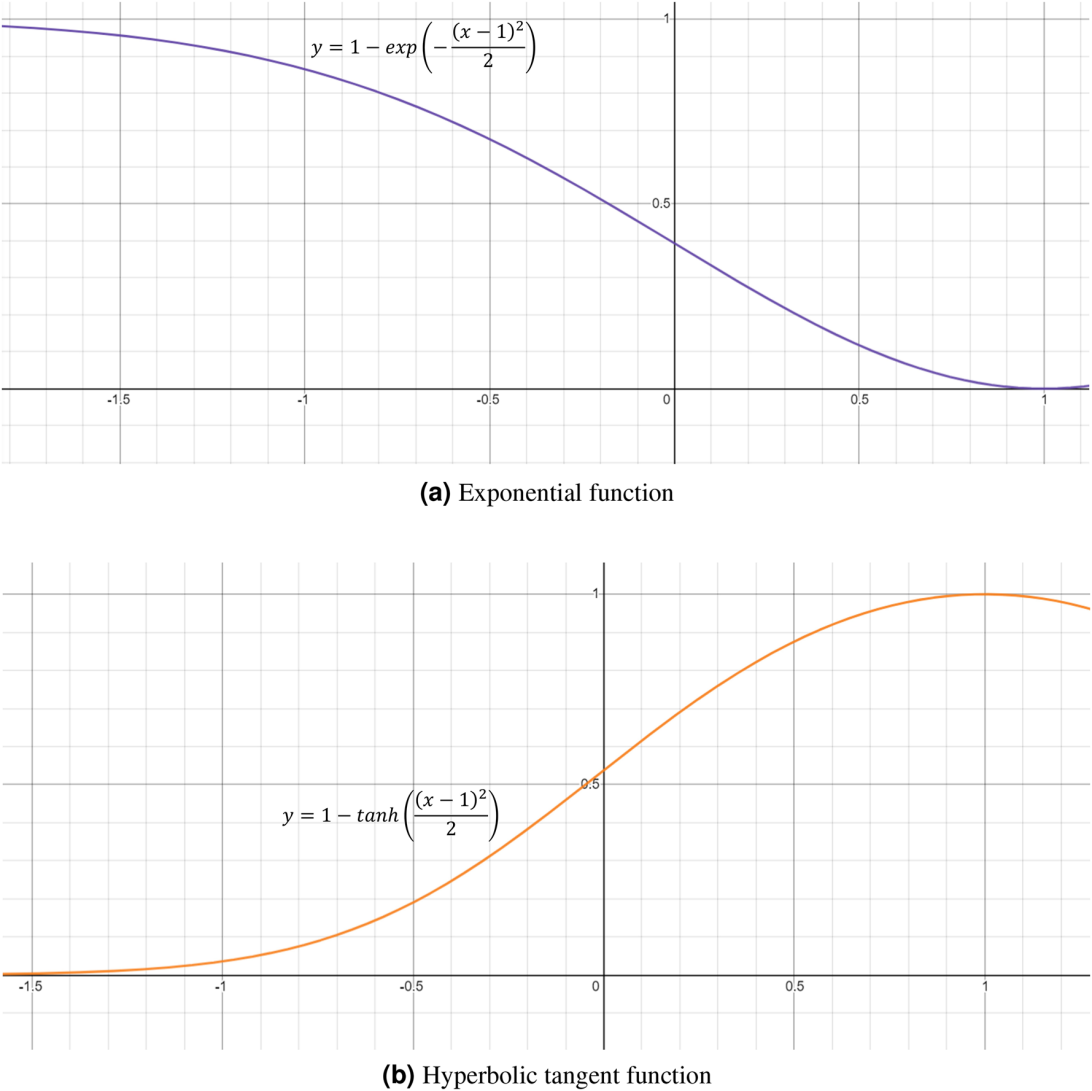
The non-linear functions used to generate fuzzy ranks in the proposed ensemble framework. *x* denotes the probability of a class of a sample data. **(a)** Quantifies the deviation from its objective for a class having prediction probability. Deviation decreases when x decreases. Eventually it becomes 0 when $\hbox {x} = 1$. **(b)** Quantifies the reward to be given to a class having prediction probability x. Reward increases when x increases. Eventually it becomes 1 when $\hbox {x} = 1$.

The fuzzy ranks are calculated by Eqs.  (2) and  (3).

2 $$\begin{aligned} R_k^{i_1}= & {} 1 - \tanh \left( \frac{(P_k^i-1)^2}{2}\right) \end{aligned}$$

3 $$\begin{aligned} R_k^{i_2}= & {} 1 - \exp \left( -\frac{(P_k^i-1)^2}{2}\right) \end{aligned}$$

The domain of definition for the functions calculating non-linear rankings will be [0, 1] as $P_k^i \epsilon [0, 1]$. The plots for these functions are shown in Fig. 5.

Equation (2) provides a reward for a classification. If x approaches 1, then the value of Eq. (2) increases i.e., the amount of reward increases. Conversely for Eq. (3), when we calculate deviation from 1, i.e., if *x* approaches 0, the deviation will be more.

Let ($RS_1^i$, $RS_2^i$, $RS_3^i$, $...$, $RS_C^i$) be the fused rank scores, where $RS_k^i$ is given by Eq. (4).

4 $$\begin{aligned} RS_k^i = R_1^{i_1} \times R_1^{i_2} \end{aligned}$$

1. $\exp (-\frac{(x-1)^2}{2})$ is concave downward in its domain of definition [0, 1] for this study. As the negative of this function is a matter of concern, it will be concave upward. Because of its negative gradient in [0, 1], the output rank score will try to shift towards 1.
2. $\tanh (\frac{(x-1)^2}{2})$ is concave upward in its domain of definition [0, 1] for this study. As the negative of this function is a matter of concern, it will be concave downward. Because of its positive gradient in [0, 1], the output rank score will try to shift towards 0.

The rank score is the product of reward and deviation for a particular confidence score obtained from a base learner. As the range of Eq. (3) is less than the range of Eq. (2), the nature of the product will be governed by Eq. (3). Lesser deviation calculated from the confidence score implies a lesser rank score. Finally, the rank scores are the only matter of concern for calculating the fused scores.

This $RS_k^i$ will signify how confidence level towards a particular class as this is the product of fuzzy ranks generated by the two different types of functions. Now the fused score tuple is ($FS_1, FS_2, FS_3, ..., FS_C$), where $FS_k$ is given by Eq. (5).

5 $$\begin{aligned} FS_k = \sum _{i=1}^{L}RS_k^i, \,\, \forall k = {1, 2 , ... , C} \end{aligned}$$

This fused score can be realized as the final score corresponding to each class. We then find the class which has the least fused score and consider it as the winner using Eq. (6). The computational complexity for the fusion strategy is $O(\text {number of classes})$.

6 $$\begin{aligned} class(I) = \min _{\forall k } FS_k \end{aligned}$$

**Figure 6:**
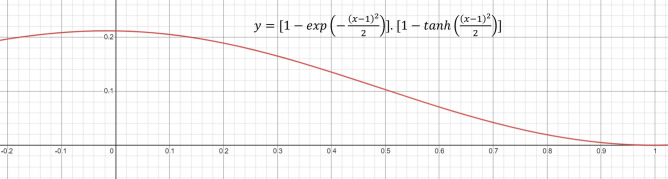
Plot of the product of the rank generating functions used in the proposed method. *x* denotes the prediction probability of a class and *y* represents the fuzzy rank product.

**Figure 7:**
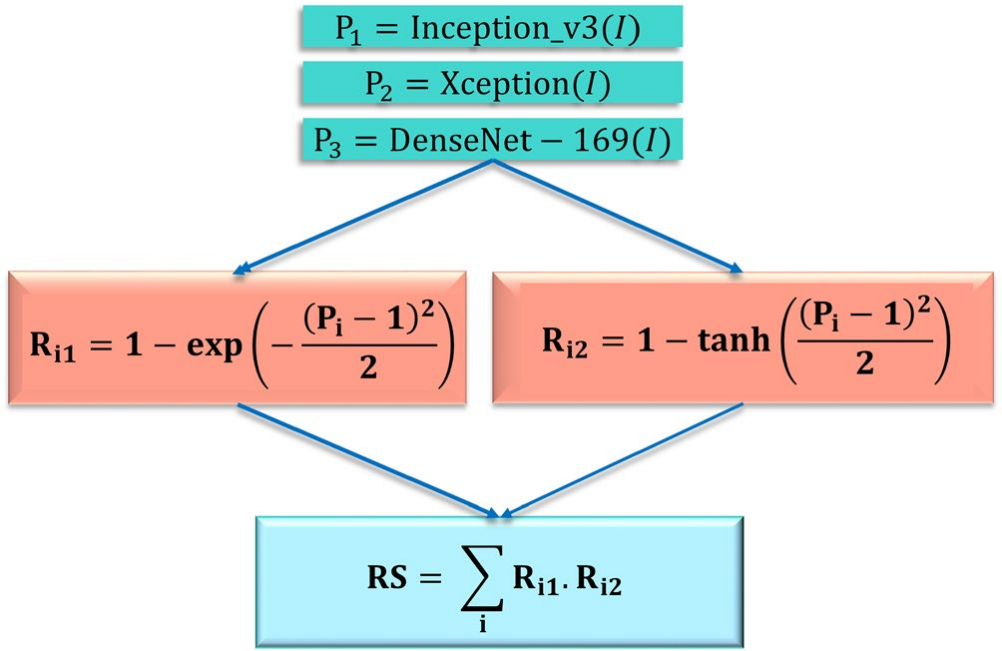
Mathematical steps of the proposed ensemble method using three CNN base models. *I* represents the input images; *P* represents the decision scores generated by the base learner and *i* represents the base learners: Inception v3 ($i=1$), Xception ($i=2$) and DenseNet-169 ($i=3$) (image has been made by R.K. using Google Slides).

From the plot of the product of two rank generating functions, shown in Fig. 6, it is clear that the final rank decreases with an increase in confidence (probability) score, which is proof of correctness. The flow diagram of the proposed ensemble method is shown in Fig. 7.

**Figure 8:**
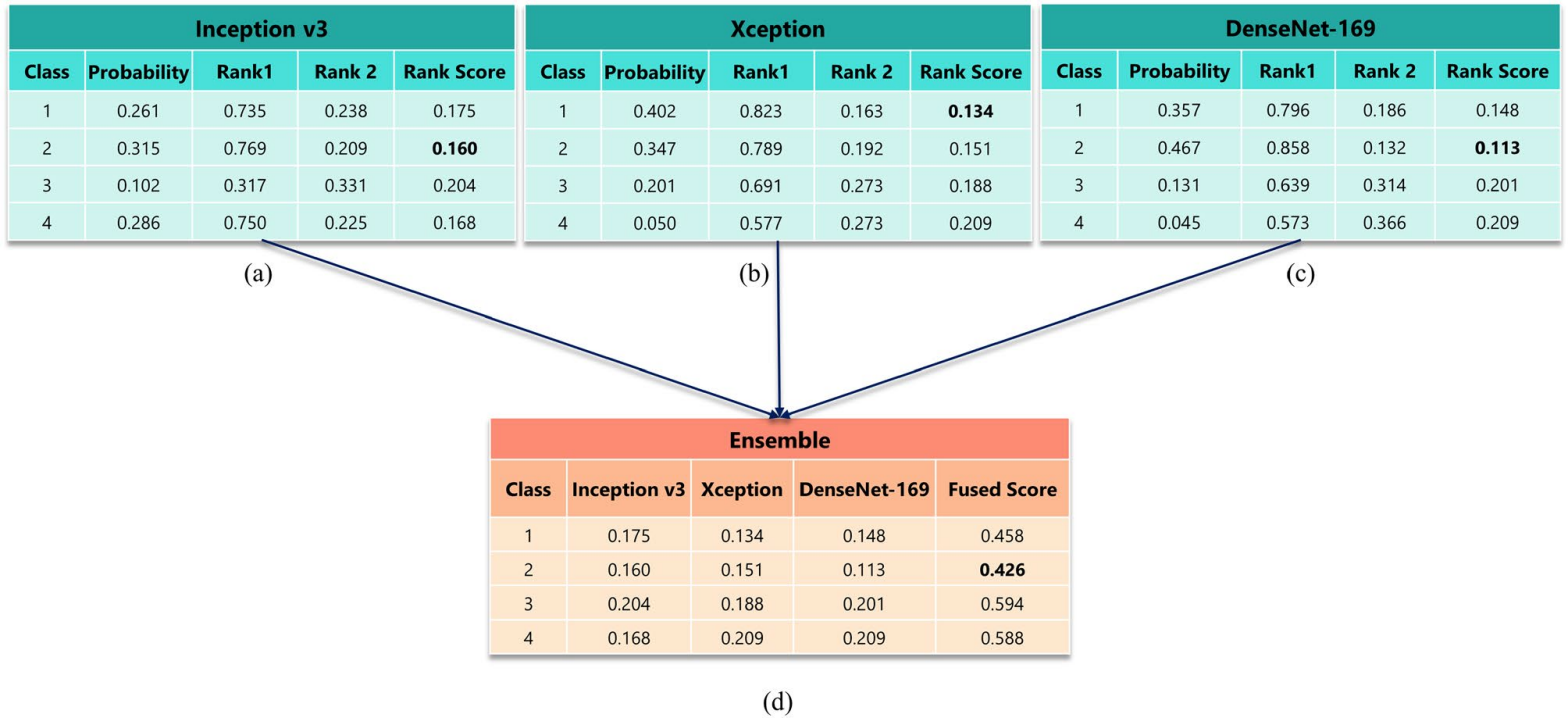
A hypothetical example showing the working procedure of the proposed ensemble model for an image taken from the 4-class dataset. Tables **(a–c)** show all the necessary calculations required to generate the rank scores from the base learners. Table **(d)** shows the overall fused score and the final decision to get the classification result. Bold font represents the rank score of the class that is declared the winner by the respective model (image has been made by R.K. using Google Slides).

Figure 8 shows an example of the proposed method for an image from the Mendeley LBC dataset (4-class). Here for an image belonging to class 2, we collect the probability values from the three base learners for each of the four classes, shown in Fig. 8a–c respectively. The probability value belonging to class 1 given by Inception v3 is 0.261. So the corresponding ranks are 0.735 and 0.238 as obtained from Eqs. (2) and  (3). Essentially the rank score becomes 0.175 by Eq. (4). Similarly, we calculate rank scores for each of the three base learners for four classes. We get 0.175, 0.134 and 0.148 as the rank scores for class 1 from Inception v3, Xception and DenseNet-169 respectively. The fused score becomes 0.458 by Eq. (5). Similarly 0.426, 0.594, and 0.588 (refer to “Fused Score” column of Table (d) of Fig. 8) are the fused scores for classes 2, 3 and 4 respectively. We can see that the winner made by Inception v3 and DenseNet-169 is class 2, but by Xception it is class 1. Here our fusion method works properly and makes a robust decision. The overall fused score is minimum for class 2, so by Eq. 6, the predicted class is 2, which is mentioned at the beginning of this explanation.

## Results and discussion

In this section, we have reported the results by evaluating the proposed ensemble model on two publicly available datasets and discussed the significance of the results obtained. We have also compared the performance of the proposed model with many existing methods to ensure the superiority of the proposed method.

### Dataset description

In the current research, we have used two publicly available benchmark datasets, namely, the Mendeley Liquid Based Cytology (LBC) dataset proposed by Hussain et al.^18^ and the SIPaKMeD Pap Smear dataset proposed by Plissiti et al.^9^ to evaluate the performance of the proposed ensemble framework.

#### Mendeley liquid based cytology dataset

**Figure 9:**
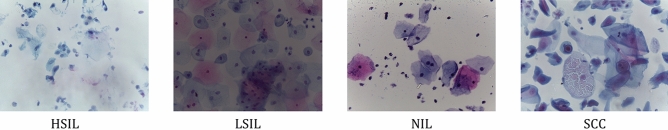
Examples of images from the Mendeley LBC dataset^18^. *HSIL* high squamous intra-epithelial lesion, *LSIL* low squamous intra-epithelial lesion, *NIL* negative for intra-epithelial lesion, *SCC* squamous cell carcinoma.

The Mendeley LBC dataset^18^ contains 963 images unevenly distributed among four classes. The images were prepared by the liquid-based cytology technique using cells obtained from 460 patients. The distribution of the images in the dataset is shown in Table 2 and some examples images from the dataset are shown in Fig. 9.

**Table 2:** Distribution of images in the Mendeley LBC smear dataset.

Class	Category	Number of images
1	Negative for intra-epithelial malignancy	613
2	High squamous intra-epithelial lesion	113
3	Low squamous intra-epithelial lesion	163
4	Squamous cell carcinoma	74
–	Total	963

#### SIPaKMeD pap smear dataset

**Figure 10:**
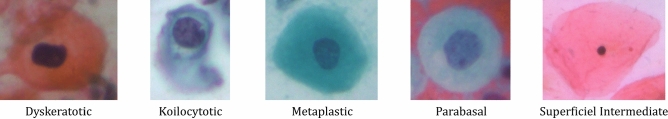
Examples of images from the SIPaKMeD Pap Smear dataset^9^.

The SIPaKMeD pap smear dataset^9^ consists of 4049 isolated cervical cell images. The cells are unevenly distributed among five different classes, classified by the experts. Normal cells are divided into two categories, namely “Superficial-Intermediate” and “Parabasal”, while abnormal (but not malignant) cells are categorized into “Koilocytes” and “Dyskeratotic”, and the final category is benign or “Metaplastic” cells. The distribution of images in the dataset is shown in Table 3 and some examples of images from the dataset are shown in Fig. 10.

**Table 3:** Distribution of images in the SIPaKMeD pap smear dataset.

Class	Category	Category	Number of images
1	Normal	Superficial-intermediate	831
2	Normal	Parabasal	787
3	Abnormal	Koilocytotic	825
4	Abnormal	Dyskeratotic	813
5	Benign	Metaplastic	793
–	–	Total	4049

### Evaluation metrics

To validate the performance of the proposed model, we have used four popular evaluation criteria: Accuracy, Precision, Recall and F1-Score. In a binary classification problem, suppose the two classes are: positive and negative. *True Positive (TP)* refers to a sample belonging to the positive class, being classified correctly. *False Positive (FP)* refers to a sample belonging to the negative class but classified to be belonging to the positive class. Similarly, *True Negative (TN)* refers to a sample being classified correctly as belonging to the negative class. *False Negative (FN)* refers to a sample belonging to the positive class but classified as being part of the negative class. Now, extending these measures to a multi-class problem with say *N* classes generates a confusion matrix, say *C*, in which the columns represent the true class and rows represent the predicted class.

The mathematical expressions of the evaluation metrics obtained from the confusion matrix *C* are thus given by Eqs.  (7),  (8), (9) and  (10).

**Accuracy:**

7 $$\begin{aligned} Accuracy =\frac{\sum _i{C_{ii}}}{\sum _i{\sum _j{C_{ij}}}} \end{aligned}$$

**Precision:**

8 $$\begin{aligned} Precision = \frac{\sum _i{C_{ii}}}{\sum _i{\sum _j{C_{ji}} }} \end{aligned}$$

**Recall or Sensitivity:**

9 $$\begin{aligned} Recall = \frac{\sum _i{C_{ii}}}{\sum _j{C_{ij}}} \end{aligned}$$

**F1-Score:**

10 $$\begin{aligned} F1-Score = \frac{2}{\frac{1}{Precision}+\frac{1}{Recall}} \end{aligned}$$

### Implementation

**Figure 11:**
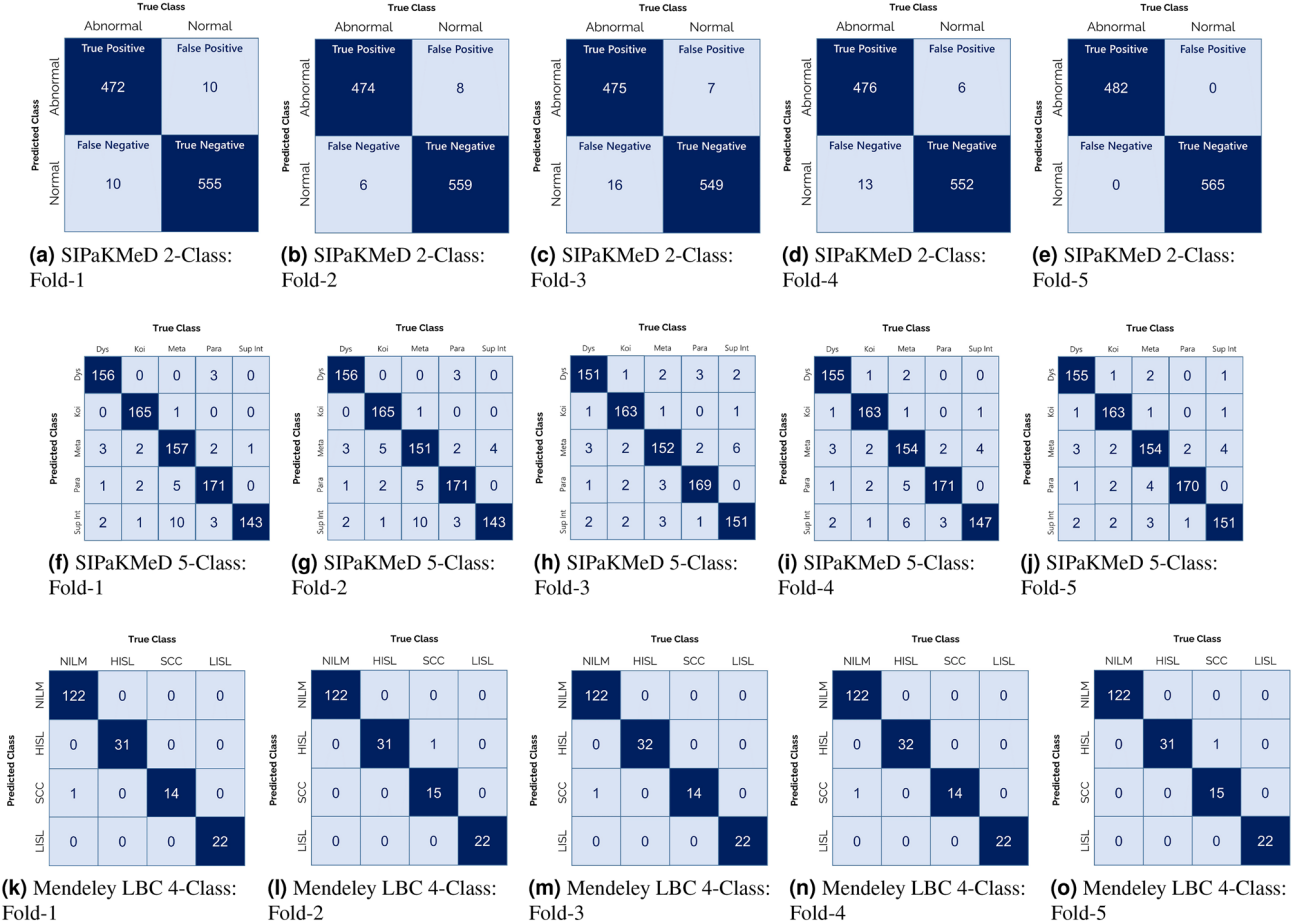
Confusion matrices obtained by the proposed method on the three datasets used in this research on fivefold cross-validation. *Dys* dyskeratotic, *Koi* koilocytotic,*Meta* metaplastic, *Para* parabasal, *SupInt* superficiel intermediate. *NILM* negative for intra-epithelial malignancy, *HSIL* high squamous intra-epithelial lesion, *SCC* squamous cell carcinoma, *LSIL* low squamous intra-epithelial lesion (images have been made by R.K. using Google Slides).

Table 4 shows the results obtained by the proposed ensemble framework on the publicly available datasets used in this work on the 5-fold cross-validation experimental setting. The results confirm that the proposed model achieves high classification accuracy and sensitivity, while also being much faster than the current manual screening procedure justifying the reliability of the automated approach. The training time per fold is 90 min for the SIPaKMeD Pap Smear dataset, and 25 min for the Mendeley LBC dataset. The confusion matrices obtained by the proposed framework on fivefold cross-validation on all the datasets used in this study are shown in Fig. 11. For the SIPaKMeD 2-class and Mendeley LBC datasets, the false positive and false negative rates for each class are fairly low. In the SIPaKMeD 5-class dataset, however, a significant number of samples are misclassified. This is more prominent for the “Superficial Intermediate” class where many samples are classified as belonging to class “Metaplastic”.

**Table 4:** Results obtained by the proposed framework on the three publicly available datasets used in this study, considering fivefold cross-validation scheme.

Dataset	Fold	Accuracy (%)	Precision (%)	Recall (%)	F1-Score (%)
SIPaKMeD 2-Class	1	98.09	98.13	98.03	98.08
	2	98.67	98.75	98.54	98.63
	3	97.80	97.85	97.73	97.79
	4	98.19	98.11	98.29	98.20
	5	100.00	100.00	100.00	100.00
	${Avg} \pm {Std. Dev.}$	${{98.55}} \pm {{0.78}}$	${{98.57}} \pm {{0.77}}$	${{98.52}} \pm {{0.79}}$	${{98.54}} \pm {{0.78}}$
SIPaKMeD 5-Class	1	95.60	95.60	95.73	95.66
	2	94.84	94.56	94.60	94.58
	3	95.34	95.21	95.34	95.27
	4	95.41	95.34	95.41	95.37
	5	95.96	96.00	95.81	95.90
	${{Avg}} \pm {{Std. Dev.}}$	${{95.43}} \pm {{0.36}}$	${{95.34}} \pm {{0.48}}$	${{95.38}} \pm {{0.43}}$	${{95.36}} \pm {{0.45}}$
Mendeley LBC	1	98.96	98.96	98.96	98.96
	2	99.48	99.12	99.48	99.30
	3	99.12	98.96	99.12	99.04
	4	99.12	99.12	99.12	99.12
	5	99.48	99.48	99.48	99.48
	${{Avg}} \pm {{Std. Dev.}}$	${{99.23}} \pm {{0.21}}$	${{99.13}} \pm {{0.19}}$	${{99.23}} \pm {{0.21}}$	${{99.18}} \pm {{0.19}}$

Avg average of the fivefolds, Std. Dev. standard deviation.

To justify the choice of the base learners, we have performed experiments using combinations of several base learners: Inception v3, Xception, DenseNet-121, DenseNet-169, DenseNet-201, VGG-16, VGG-19, ResNet-50 and ResNet-101. The results obtained are reported in Table 5. The proposed combination of Inception v3, Xception and DenseNet-169 obtains the best result on all the three datasets and is significantly better than the second-best performance obtained by the ensemble of Inception v3, VGG-16 and DenseNet-169. The performance of an ensemble depends more upon the ability of the base learners to provide complementary information, than the individual performance of the base learners. Clearly, the three classifiers used in this research are better suited for the ensemble than the other tested combinations.

The proposed framework can be used as a plug-and-play model where new test images can be passed through the model to generate the predictions through the ensemble scheme, and this will eventually help the expert clinicians to make a quicker and accurate decision. For testing on new test samples, about 5 seconds are required per image. So, the proposed CAD method is reliable for use in the field.

**Table 5:** Results obtained on ensembling various combinations of base learners on all the three datasets used in this study.

Model-1	Model-2	Model-3	Ensemble result (classification accuracy %)		
			Mendeley LBC	SIPaKMeD 2-Class	SIPaKMeD 5-Class
Inception v3	Xception	DenseNet-121	96.05	95.38	92.30
Inception v3	Xception	DenseNet-201	94.04	93.89	90.60
Inception v3	VGG-16	DenseNet-169	97.37	96.39	93.01
Xception	VGG-16	ResNet-50	95.06	93.98	91.05
DenseNet169	VGG-19	ResNet-50	96.36	94.68	91.56
DenseNet169	VGG-19	ResNet-101	95.64	93.07	90.42
Inception v3	Xception	DenseNet-169	99.23	98.55	95.43

**Figure 12:**
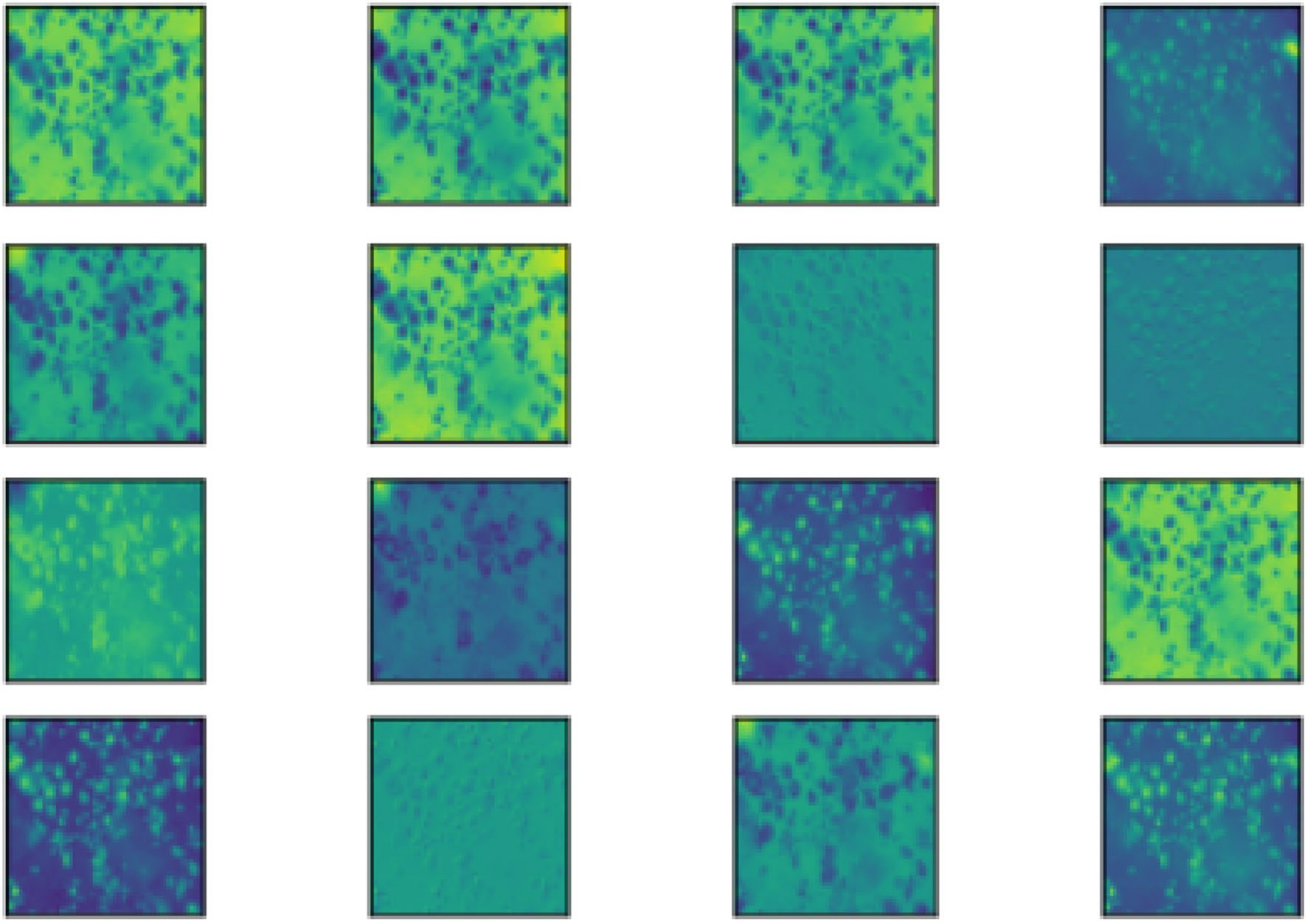
Visualization of the convolution filters of the Inception v3 model on the Mendeley LBC dataset (the plots have been formed using Keras framework of Python).

All the base models are generated by customizing the pre-trained models, and all the pre-trained models have a sufficient number of convolution layers. Hence, we do not require to add more convolution layers in our customized models. However, for the visualization purpose, we have provided the filters of convolution for the Inception v3 model on the Mendeley LBC dataset in Fig. 12.

**Figure 13:**
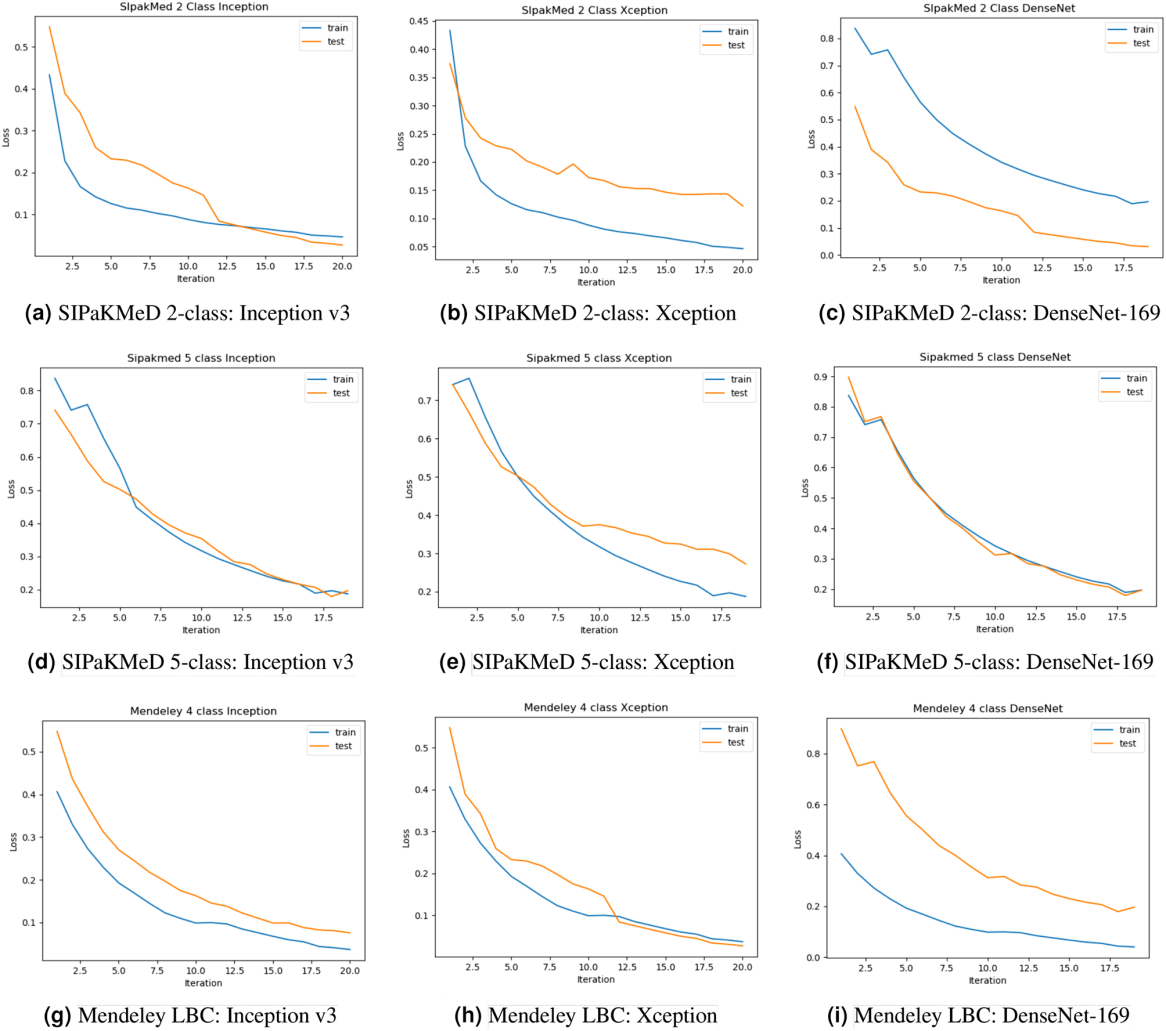
Loss curves obtained on fine-tuning the three CNN base learners: Inception v3, Xception and DenseNet-169 on the three datasets used in this research—**(a–c)** SIPaKMeD 2-class dataset, **(d–f)** SIPaKMeD 5-class dataset and **(g–i)** Mendeley LBC 4-class dataset (The loss curves have been plotted using Keras framework of Python).

### Robustness of base learners

It is evident from Table 6 that our model performs well in all the datasets we have tested on. To prove that the model is not overfitted even after being trained on a smaller dataset, we have provided loss curves Fig. 13 for base learners. A decrease in the validation loss along with training loss is prominent in the provided loss curves for the base learners. It indicates that the base learners we have fine-tuned perform robustly and are not overfitted.

### Comparison to state-of-the-art

**Figure 14:**
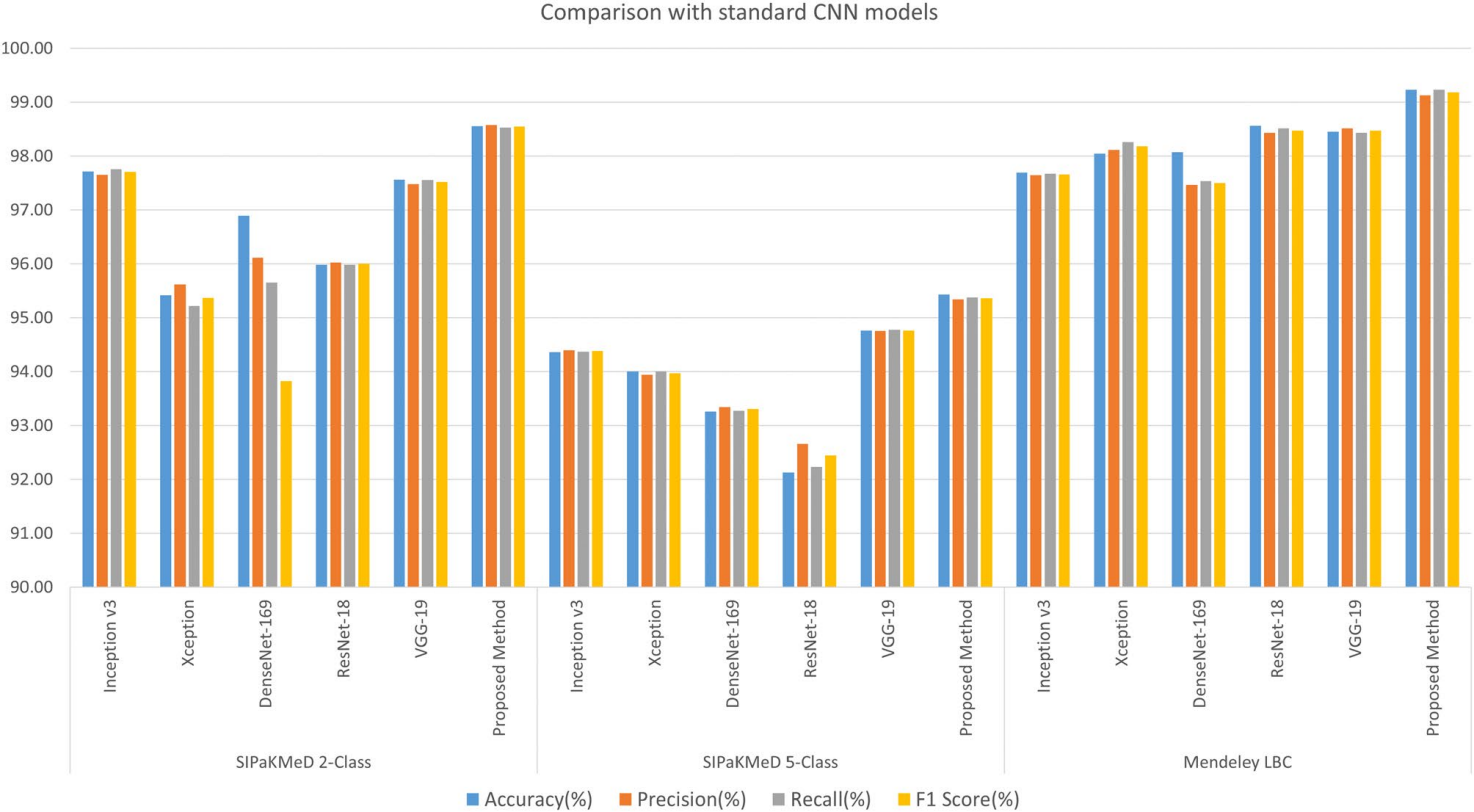
Comparison of the proposed ensemble model with some standard CNN models in literature: Inception v3^4^, Xception^5^, DenseNet-169^6^, ResNet-18^22^, VGG-19^23^ (image has been made by R.K. using Google Sheets).

Table 6 shows the classification results obtained by the base classifiers and their ensemble using the proposed ensemble technique. In the SIPaKMeD Pap Smear dataset, the Inception v3 model performs better than the Xception and DenseNet-169 models, whereas, the Xception model performs better than the other two in the Mendeley LBC dataset. The proposed ensemble method performs significantly better than all the base classifiers in both datasets. This indicates that the classification capability of different CNN models has some dependency upon the dataset under consideration: Inception v3 performs better for single-cell images dataset, while Xception performs better for the whole slide images dataset; but the proposed ensemble method performs robustly by considering the confidence score from all its base learners. Thus the ensemble model can be generalized better than a single CNN classifier. Figure 14 shows the results of some standard CNN models obtained on the datasets, compared to the proposed ensemble framework.

**Table 6:** Comparison of the classification performance of the base learners and their ensemble using the proposed scheme.

Dataset	Method	Accuracy(%)	Precision (%)	Recall (%)	F1-Score (%)
SIPaKMeD 2-Class	Inception v3	97.71	97.65	97.75	97.70
	Xception	95.42	95.61	95.22	95.37
	DenseNet-169	96.89	96.11	95.65	93.82
	Proposed ensemble	98.55	98.57	98.52	98.54
SIPaKMeD 5-Class	Inception v3	94.36	94.40	94.37	94.38
	Xception	94.00	93.94	94.00	93.97
	DenseNet-169	93.26	93.34	93.27	93.30
	Proposed ensemble	95.43	95.34	95.38	95.36
Mendeley LBC	Inception v3	97.69	97.64	97.67	97.65
	Xception	98.04	98.11	98.26	98.18
	DenseNet-169	98.07	97.47	97.53	97.50
	Proposed ensemble	99.23	99.13	99.23	99.18

**Table 7:** Comparison of the proposed framework with some state-of-the-art methods on the SIPaKMeD pap smear dataset.

Dataset	Method	Accuracy (%)	Precision (%)	Recall (%)	F1-Score (%)
SIPaKMeD 2-Class	Win et al.^13^	98.27	–	–	–
	Proposed method	98.55	98.57	98.52	98.54
SIPaKMeD 5-Class	Plissiti et al.^9^	95.35	–	–	–
	Win et al.^13^	94.09	–	–	–
	Sevi et al.^21^	88.40	–	–	–
	Proposed method	95.43	95.34	95.38	95.36

**Figure 15:**
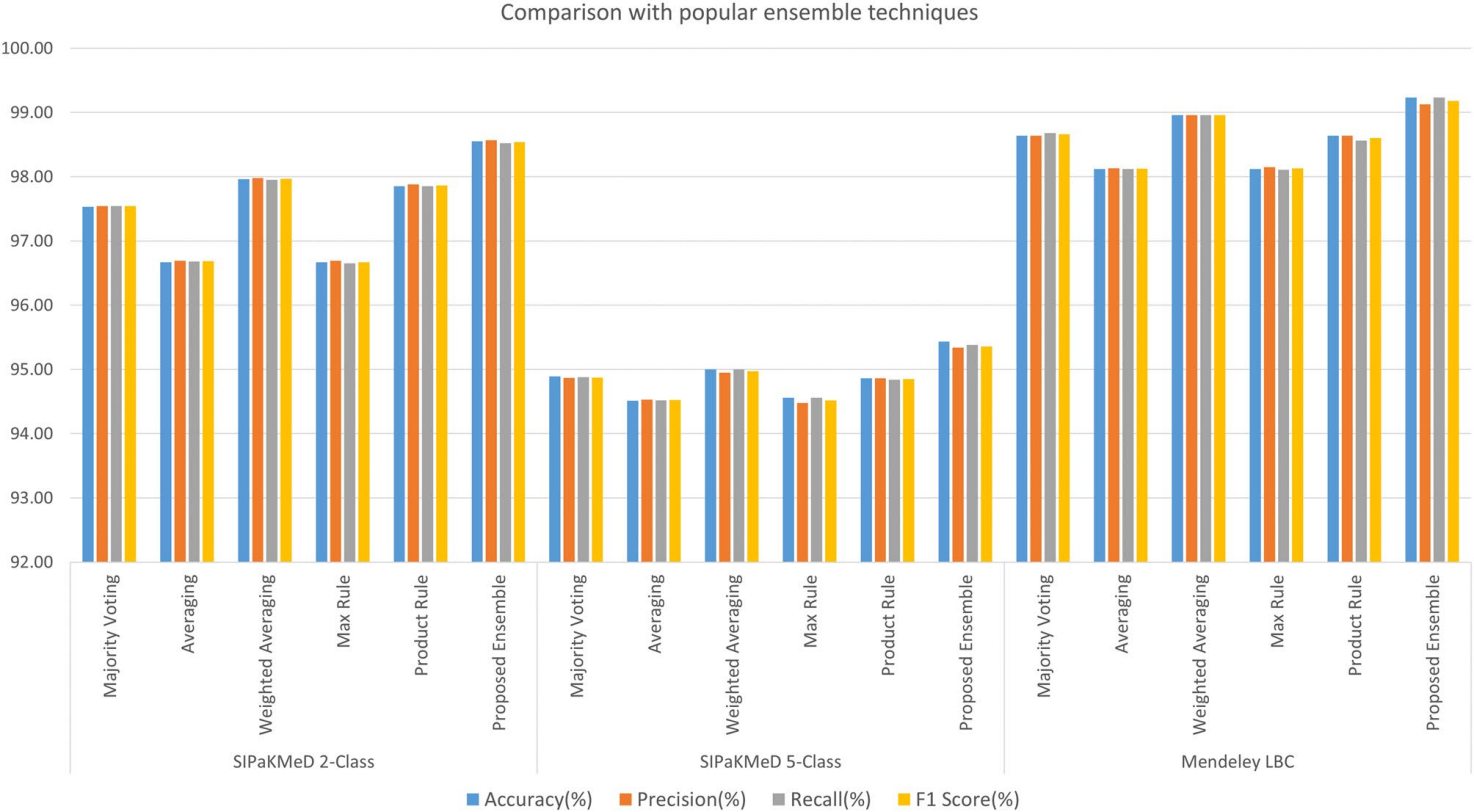
Comparison of the proposed ensemble model with some popular fusion techniques in literature using the same base learners: Inception v3, Xception and DenseNet-169 (image has been made by R.K. using Google Sheets).

Some fusion schemes are popularly used in literature, like majority voting, probability averaging, and weighted probability averaging, etc. Figure 15 shows the comparison of the proposed ensemble scheme to some of these popular ensemble techniques that have been used in literature, using the same base classifiers: Inception v3, Xception and DenseNet-169. In both datasets, the weighted probability averaging technique gives classification results closest to the proposed ensemble technique, wherein the weights have been determined experimentally. But, this is a static process, since, after the selection of the weights, there is no scope for dynamically refactoring the weights at prediction time. The proposed ensemble model, however, assigns ranks to the classifiers on each test sample based on the confidence in predictions by the base learners, which leads to superior classification performance.

Table 7 compares the proposed approach with some state-of-the-art results on the datasets. No published work has been found on the Mendeley LBC dataset at the time of writing this manuscript for comparison.

### Error analysis

**Figure 16:**
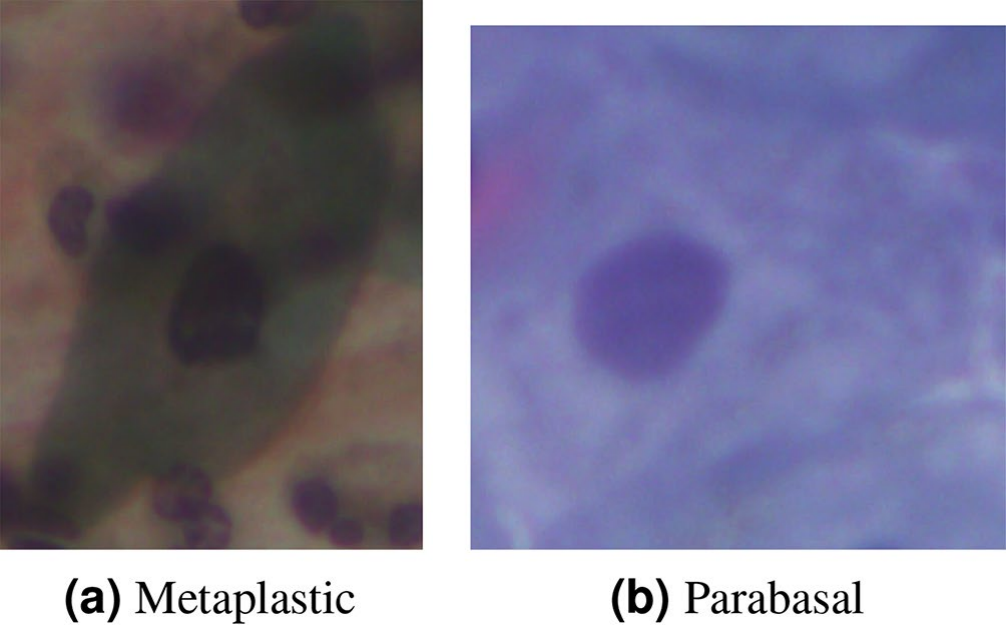
Examples of test samples from the SIPaKMeD Pap Smear dataset^9^ where one or more of the base classifiers predict incorrectly, but the ensemble predicts correctly. **(a)** DenseNet-169 classifies the sample as: “Koilocytotic” with confidence 31%, Xception classifies the sample as: “Parabasal” with confidence 36% and Inception v3 classifies the sample as: “Metaplastic” with confidence 98%. Ensemble prediction is: “Metaplastic”. **(b)** DenseNet-169 classifies the sample as: “Koilocytotic” with confidence 32%, Xception classifies the sample as “Parabasal” with confidence 95%, and Inception v3 classifies the sample as “Parabasal” with confidence 98%. Ensemble prediction is: “Parabasal”.

Figure 16 shows some examples from the SIPaKMeD Pap Smear dataset where one or more base classifiers made wrong predictions on the sample, but the ensemble made the correct predictions. Figure 16a is a sample from the “Metaplastic” class of the SIPaKMeD dataset, which is classified as “Koilocytotic” by the DenseNet-169 with the confidence of 31%, and “Parabasal” by the Xception model with the confidence of 36%. However, being classified as “Metaplastic” by the Inception v3 model with 98% confidence allowed the ensemble to predict the sample correctly. Similarly, the sample in Fig. 16b, originally of class “Parabasal” is misclassified as “Koilocytotic” by the DenseNet-169 model with the confidence of 32% while the Xception and Inception v3 models predicted correctly with confidence scores of 95% and 97% respectively, thus allowing the ensemble to predict the sample correctly as “Parabasal”. Figure 16a has multiple nuclei in its image and the cytoplasm in Fig. 16b is not distinguishable. Although both the test samples had a bad image quality, the proposed framework was able to correctly classify them, justifying the robust performance of the model.

**Figure 17:**
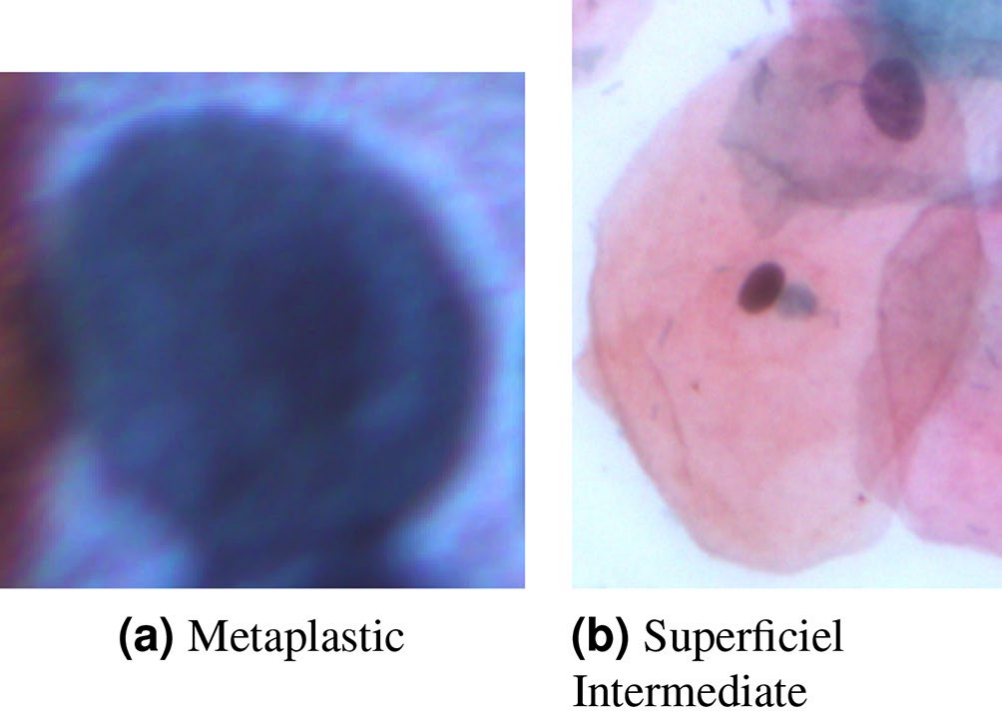
Examples of some misclassified samples from the SIPaKMeD Pap Smear dataset^9^. **(a)** Final prediction: “Parabasal” **(b)** Final prediction: “Koilocytotic”.

Figure 17 shows some test samples from the SIPaKMeD Pap Smear dataset that were misclassified by the proposed framework. Figure 17a shows a sample from the “Metaplastic” class which is misclassified as “Parabasal”. The nucleus in the image is not distinguishable from the cytoplasm leading to an incorrect classification by the ensemble model. Figure 17b shows an image belonging to the “Superficial Intermediate” class, but misclassified as “Koilocytotic”. The reason for this might be the intrusion of another Superficial Intermediate cell in the image on the top right corner. This unwanted cell is not completely included in the image and only part of the cytoplasm is visible. This leads to an erroneous nucleus to cytoplasm ratio, leading the framework to classify the image as a “Koilocytotic” class.

### Statistical analysis

To statistically analyse the viability of the proposed ensemble framework concerning the base learners used to form the ensemble, McNemar’s statistical test^24^ is performed. McNemar’s test is a non-parametric analysis of paired nominal data distribution. The “$p-value$” signifies the probability of two models being similar, thus, a lower $p-value$ is desired. To reject the null hypothesis that the two models are similar, the $p-value$ needs to be smaller than $5\%$ that is, if $p-value<0.05$, we can safely say that the two models under consideration are statistically different. From Table 8, it can be concluded that in both the datasets (and in both settings of the SIPaKMeD pap smear dataset), the null hypothesis is rejected, that is, the ensemble model is markedly different from the base learners.

**Table 8:** Results of the McNemar’s test performed between the proposed ensemble model and the base learners used: null hypothesis is rejected for all cases.

Dataset	Comparison model	p-value
SIPaKMeD 2-Class	Inception v3	2.15E−02
	Xception	1.80E−04
	DenseNet-169	4.30E−03
SIPaKMeD 5-Class	Inception v3	1.61E−03
	Xception	9.80E−04
	DenseNet-169	1.20E−03
Mendeley LBC	Inception v3	8.44E−04
	Xception	4.13E−02
	DenseNet-169	1.79E−04

### Additional test

To further justify the robustness of the proposed ensemble framework, we evaluate it on an 8-class colorectal cancer histopathology dataset: the Zenodo 5K dataset^25^. The distribution of images in the dataset is tabulated in Table 9.

**Table 9:** Distribution of images in the Zenodo 5K dataset used for the additional test in this research.

Class	Category	Number of images
1	Tumour epithelium	625
2	Simple stroma	625
3	Complex stroma	625
4	Immune cells	625
5	Debris	625
6	Normal mucosal glands	625
7	Adipose tissue	625
8	Background (no tissue)	625

**Table 10:** Results (accuracies in %) obtained by the proposed ensemble framework and its base classifiers on the Zenodo 5K breast histopathology dataset.

Fold	Inception v3	Xception	DenseNet-169	Proposed ensemble
1	93.12	89.34	88.80	96.90
2	94.02	88.19	87.24	96.91
3	91.20	89.41	87.49	96.90
4	95.80	89.33	89.96	96.95
5	93.60	88.18	89.60	96.86
${{Avg}} \pm {{Std. Dev}}$	${{93.55}} \pm {{1.66}}$	${{88.89}} \pm {{0.64}}$	${{88.62}} \pm {{1.22}}$	${{96.90}} \pm {{0.03}}$

**Table 11:** Comparison of the proposed ensemble method with some previous methods in literature on the Zenodo 5K dataset used for the additional test in this study.

Method	Approach	Accuracy (%)
Kather et al.^25^	Texture analysis	87.40
Tellez et al.^26^	Unsupervised stain colour normalization	79.66
Proposed	Classifier ensemble	96.90

Table 10 shows the results obtained upon evaluation using the fivefold cross-validation scheme. From the table, it can be noted that the ensemble of the classifiers yield results significantly better than its constituent base learners in this multi-class data arrangement, justifying that the proposed ensemble method is robustly boosting the performance of the base learners. Comparison of the results obtained by the proposed method and some state-of-the-art methods are tabulated in Table 11, where the proposed ensemble method is seen to outperform the previous methods by a significant margin.

## Conclusion and future work

Cervical cancer is one of the leading causes of mortality among women, whose population-wide screening is restricted due to the expensive and laborious detection process demanding the expertise of clinicians for detection. In this paper, we develop a CAD framework that classifies cytology images using an ensemble of three standard CNN based classifiers. The proposed ensemble model generates ranks of the classifiers using two non-linear functions which help to take into account the confidence in predictions of the base learners. The proposed CAD framework, when evaluating two benchmark datasets for cervical cytology classification, produces competitive results in terms of accuracy and sensitivity to the disease, thus justifying the effectiveness of the framework. The fast detection tool developed can function like a plug-and-play model that requires little intervention of the expert clinicians for cervical cancer screening, and hence suitable for incorporation in the field.

As discussed previously, some of the images could not be accurately classified by the proposed ensemble model, due to poor image contrast or the presence of overlapping cells. So there might be a need for preprocessing of the images, which we would like to address in the future. We may try contrast enhancement techniques or prior segmentation of cells for isolating overlapping cells. We may also consider ensembles of other base learners, and explore different rank generation functions to perform the ensemble.
